# Comparison of Trunk Activity during Gait Initiation and Walking in Humans

**DOI:** 10.1371/journal.pone.0008193

**Published:** 2009-12-07

**Authors:** Jean-Charles Ceccato, Mathieu de Sèze, Christine Azevedo, Jean-René Cazalets

**Affiliations:** 1 Université de Bordeaux, UMR 5227, laboratoire « Mouvement adaptation cognition », CNRS (Centre National de la recherche scientifique), Bordeaux, France; 2 Université Victor Segalen de Bordeaux 2, Laboratoire d'anatomie, EA 4136 Handicap et système nerveux-IFR8-IFR25, Bordeaux, France; 3 Service de Médecine Physique et Réadaptation, Pôle Neurosciences Cliniques, CHU (Centre Hospitalier Universitaire) Pellegrin, Bordeaux, France; 4 INRIA (Institut National de recherche en Informatique et Automatique) / LIRMM (Laboratoire d'Informatique, Robotique et Microélectronique de Montpellier), team DEMAR, Montpellier, France; Karolinska Institutet, Sweden

## Abstract

To understand the role of trunk muscles in maintenance of dynamic postural equilibrium we investigate trunk movements during gait initiation and walking, performing trunk kinematics analysis, Erector spinae muscle (ES) recordings and dynamic analysis. ES muscle expressed a metachronal descending pattern of activity during walking and gait initiation. In the frontal and horizontal planes, lateroflexion and rotation occur before in the upper trunk and after in the lower trunk. Comparison of ES muscle EMGs and trunk kinematics showed that trunk muscle activity precedes corresponding kinematics activity, indicating that the ES drive trunk movement during locomotion and thereby allowing a better pelvis mobilization. EMG data showed that ES activity anticipates propulsive phases in walking with a repetitive pattern, suggesting a programmed control by a central pattern generator. Our findings also suggest that the programs for gait initiation and walking overlap with the latter beginning before the first has ended.

## Introduction

Since the first studies on human locomotion, motor control investigations have mainly examined the lower limbs as the primary actors in locomotion, while the trunk and its control during walking has received much less attention [1], [2] [3]–[6]. In humans, the trunk represents 60% of the total body mass with its high position relative to the feet allowing it to exert an important “inverted pendulum” leverage effect [7]. However, the trunk is highly articulated and actuated by many muscles, providing it with the versatility to participate actively in the various motor tasks that humans undertake [8], while maintaining trunk balance. This versatility therefore suggests a necessity for a complex regulation of trunk movements resulting from a combination of anticipatory and reactive muscular actions.

Kinematic studies during walking have demonstrated a general inclination of the trunk in the sagittal plane, a lateroflexion on each side per cycle in the frontal plane and a phase opposition between higher and lower trunk rotations in the horizontal plane [9], [10]. Electromyographic studies have highlighted the importance of the ES in the organization of locomotor patterns during walking [11]–[13] and other various rhythmic motor tasks [12]. During forward walking, ES activity occurs mainly around the double support phase [5], [14], with a sequential organization along the spinal axis from top to bottom [12], [15]. This motor pattern presents similarities with the caudo-rostral metachronal (segment by segment) propagating patterns that have already been observed from electrophysiological and kinematic studies in newborn rats [16], [17] and lamprey [18].

The aim of the present study was to understand the role of the trunk ES muscles in dynamic postural equilibrium by comparing their activation during two different tasks, gait initiation and walking, the first being a transition between postural and dynamic stability while the second is mainly dynamically stabilized. To extend the comprehension of the role of the metachronal activation of ES during walking [12] it was necessary to link it to synchronized kinematics and dynamic data analyses. However, studying an activity such as walking presents a major drawback, since due to its cyclic nature, it is not possible to discriminate between anticipatory and reactive adjustments. Moreover to our knowledge little work has been done on the muscular and kinematic activity of the upper body during gait initiation. Therefore, in the present study, we have compared trunk activity during both gait initiation and steady state walking in order to gain further insight into 1), the role of ES activity in normal walking, and specifically, how this muscle contributes to driving trunk movement that facilitate lifting the pelvis and the leg 2), how trunk activity is programmed during a transition between postural and dynamical state like gait initiation.

## Materials and Methods

### Subjects

Nine healthy men were recruited for this study from university students and the local community. Their ages, heights and weights ranged from 23 to 42 years (27±6 years), 168 to 191 cm (179±7 cm) and 65 to 81 kg (70±6 kg), respectively. Their body mass index was inferior to 25 kg/m^2^ (21±1 kg/m^2^). We tested the leading leg dominance of each subject with two tests: (1) 3 imbalance trials, by pushing their back from a quiet stance, and (2) 3 gait initiations before mounting any equipment on them. Four subjects were left-leg dominant and five were right-leg dominant (i.e. initiating walking with the left or right leg respectively). They had no history of back pain or any disorder related to the locomotor apparatus. The subjects gave their written informed consent and the procedures were approved by the local ethics committee, Comite de protection des personnes Bordeaux A n° 2005/46, and are in accordance with the ethical standards in the Declaration of Helsinki.

### Procedure

The subjects wore shorts and sandals and were asked to walk over a delimited 11 m pathway on the floor always in the same direction. The calibration area (1.2 m×4 m centered on force plates) was in the middle of this pathway. Each subject performed: (1) 5 gait initiation trials starting with one foot on each force plate and was asked to walk at his “natural speed”, beginning with their leading leg; (2) 10 walking trials starting 4 meters ahead of the force plates and walking at a “steady” speed when he entered the recording area and reached the force plates. Between each trial the subjects rested for 3 minutes. All recording systems presented below (force plates, EMG and kinematics) were synchronized in time.

### EMG

The protocol for ES muscle EMG recordings was the same as described previously (de Seze et al. 2008). Tripolar surface electrodes (two poles for signal and one pole for mass, Thought Technology, Canada, 10 mm diameter, 10 mm inter-electrode distance) were connected to EMG recording units (KineMyo units, Kine ehf, Iceland). In the present study the following muscles were recorded: *Erector Spinae* (ES) monitored bilaterally at various spine levels (C7, T3, T7, T12, L3) according to previous observations. In accordance with previous studies on gait initiation, we recorded the *gastrocnemius* (GN) of the first stance leg and *tibialis anterior* (TA) of the first swing leg in the initiation step. These muscles are the first to be activated during gait initiation[19].

### Kinematics

Trunk and other body part movements were recorded using a 3D optical motion capture system (Elite, BTS, Italy). We used 8 cameras (sampling frequency of 100 Hz) to have a calibrated area of 3.5×1.2×1.8 m^3^ and a system-announced precision of 2 mm position error. We used spherical reflecting passive markers 15 mm in diameter. Markers were placed on the spinous processes of the C7, T3, T7, T12, L3 and S1 vertebra. Other markers were placed on the sternal notch, shoulders (acromions), wrists, antero-superior iliac spines, trochanters, knees, ankles and three markers on the head (2 temporal and occiput).

Data were processed using the Biomech software (BTS, Italy) to reconstruct the marker positions. Space calibration was oriented such that the X-axis was aligned with the walking direction, the Y-axis was vertical and the Z-axis was transversal to walking direction in the subject from left to right According to this orientation, the planes used to study kinematic data were defined as follows: sagittal plane (XY), frontal plane (YZ) and horizontal plane (XZ).

### Dynamics

3D forces and torques under each foot were recorded during the initiation step and during one cycle for each walking trial at 1kHz. The force plates (AMTI, USA) were spatially calibrated together with the Elite system to assess the center of pressure (COP) position in the same reference than kinematics data. The position of the COP was calculated during gait initiation and walking as the barycenter of each plate COP (maximum one foot per force plate at all time), balanced with the corresponding resultant forces.

### Data Analysis

EMGs were high-pass filtered at 35 Hz with Kineworks software (Kine ehf, Iceland), and then rectified, smoothed with moving average window algorithm and time-normalized in cycles with a custom-made routine under Scilab software (INRIA, France). The instants of maxima and beginning of bursts were detected in each cycle using AxographX software (AxoGraph Scientific, Australia) and then averaged.

Kinematic data were smoothed and processed with Smart Analyzer (BTS, Italy) for time normalization and to calculate average positions, distances and angles. We defined direction of angles in each plane, so that the “positive direction” was X to Y in the sagittal plane, Z to Y in the frontal plane and Z to X in the horizontal plane. In order to compare right-leg and left-leg dominant subjects in the gait initiation task, we performed symmetry on angles (around 0° or 180° considering cases) in the frontal and horizontal plane and on EMGs (inversion of left and right EMGs) of left-leg dominant subjects. This allowed us to combine left-leg and right-leg dominant subjects for averaging.

These data were then exported in text files for graphic design with Igor Pro (Wavemetrics, USA).

### Gait Initiation and Walking Cycle Definition

**Figure 1:**
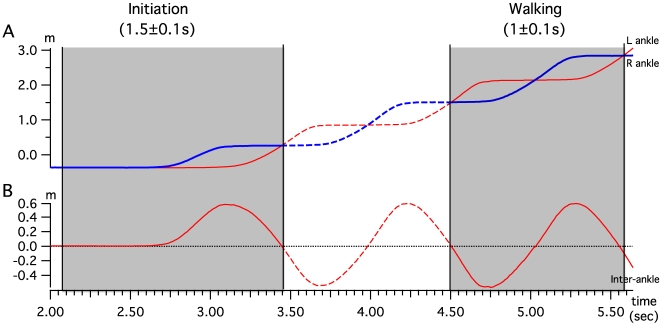
Gait initiation and walking cycle parameters: definition. A) Trajectory of left and right ankle displacement in the forward direction for gait initiation and walking. The beginning and end of the cycle are set when the two curves cross each other in the same direction. B) Inter-ankle distance in the forward direction for gait initiation and walking. The beginning and end of the cycle are set when the curve crosses the 0 in the same direction.

To average trials and compare gait initiation and walking, we first defined the normalized cycles. Each individual cycle was extracted from locomotor sequences using an index based on kinematic and dynamic data. As shown in Figure 1A, a highly reproducible and easy to use kinematic event was the “ankle crossing” instant (ankles in front of each other in sagittal plane) in the forward direction (X-axis). To automatically detect this event, we used the inter-ankle distance in the X direction (Fig. 1B) as the reference signal, and “ankle-crossing” instants were located when this signal was equal to zero. The walking cycle was then defined by two successive zero crossings of the inter-ankle distance in the same direction (up or down). The other ankle crossing for each walking cycle occurred at 50% of the cycle.

**Figure 2:**
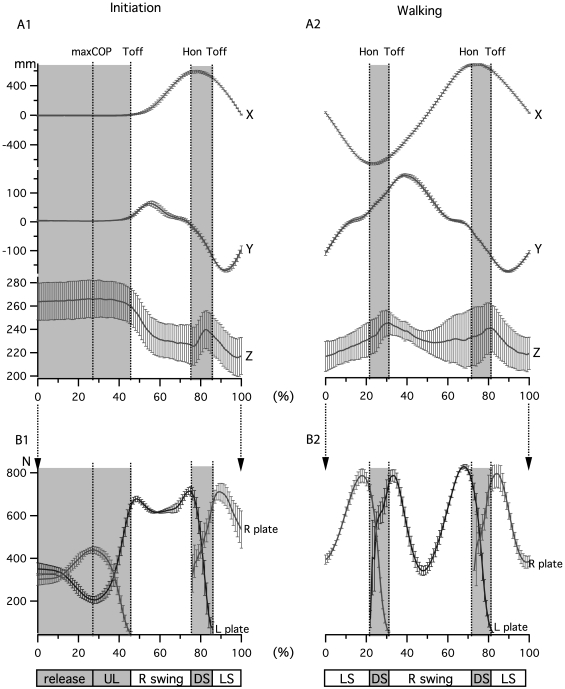
Definition of main events from inter-ankle distance and force plate during gait initiation and walking. A) Mean±SD inter-ankle distance on the three axes of space during gait initiation and walking in one subject. B) Mean±SD of vertical resultant force along Y axes on each force plate (R, Right, L, Left) allowing definition of events and phases of gait initiation and walking cycle.

For gait initiation, the end of the period was defined by the “ankle crossing”. After the first toe off, the first swinging ankle raised vertically (Y-axis) more than it moved forward (X-axis) (Fig. 2A1). From this “ankle-crossing” time point, the middle of gait initiation was set at the first toe off (exactly 45%) and using a pre-trigger, the total cycle was defined from 0%. This allowed us good comparison of first step gait initiation and walking cycle, while providing sufficient time before first toe off to include all the main events occurring during the preparatory phase of gait initiation, as shown below.

As illustrated in Figure 2 (bottom bars), the sub-phases between two events were defined as follows: release from T0 to maxCOP, unloading (UL) from maxCOP to first toe off, right or left swing (R swing or LS, white areas) from one toe off to the next heel on and double support (DS, shaded areas) between heel on and toe off [20].

## Results

### Gait Initiation and Walking Cycle Parameters

For all subjects, the mean duration for gait initiation phase was 1.5±0.1 s and walking cycle was 1±0.1 s with step length of 0.7±0.2 m. The “natural walking speed” calculated from these values was 1.4±0.2 m.s^−1^ (5.1 km.h^−1^). Using the method described above for temporal cycle normalization, we identified EMG, kinematic and dynamic signals for averaging and statistical analysis.

Figures 2A1 and 2A2 present the mean (±SD) inter-ankle distance on the three axes for one subject. Figures 2B1 and 2B2 present corresponding force plate data allowing the determination of the main events and phases of gait initiation and walking. Double support started with a heel-on and ended with a toe-off during normal walking. The main events mean timings during gait initiation and walking are reported Table 1. From these data, we established that the first double support phase represents at mean 11% (220 ms) of gait initiation phase according to our definition of gait initiation, and 10% (100 ms) of the walking cycle, which is in agreement with the literature [21].

**Table 1:** Mean±SD timings of foot contacts, events and phases during gait initiation and walking.

A. Gait initiation						
Events	T0	maxCOP	1^st^ Toff	1^st^ Hon	2^nd^ Toff	ankle crossing
Timings	0%	27.5±3.3%	45%	75.3±1.6%	86.4±1.3%	100%
Sub-phases	release	unloading(UL)	R swing	DS	L swing	
B. Walking						
Events	ankle crossing	Hon	Toff	Hon	Toff	ankle crossing
Timings	0%	21.2±0.9%	31.4±0.9%	21.5±0.7%	81.7±0.7%	100%
Sub-phases	L swing	DS	R swing	DS	L swing	

### Gait Initiation and Walking Legs Characteristics

**Figure 3:**
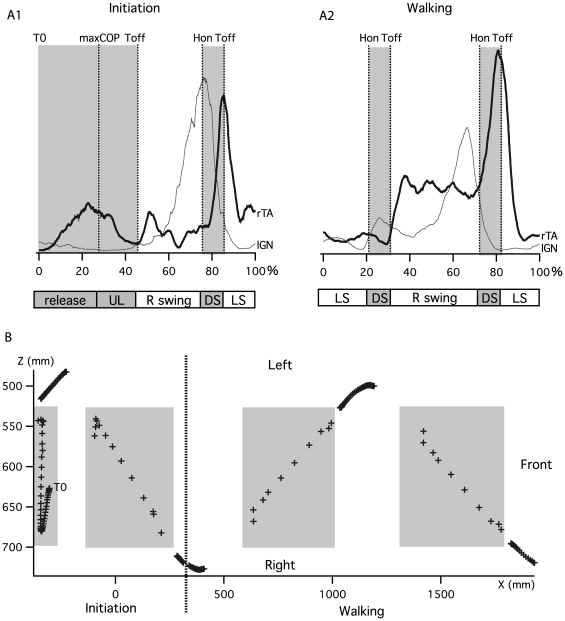
Leg muscle EMGs and center of pressure (COP) trajectory. A) Right *tibialis anterior* (rTA) anf left *gasctrocnemius* (lGN) electromyographic activity during gait initiation and walking for one trial and one subject. B) COP trajectory reconstruction obtained from the force plate signal during one trial of gait initiation and one trial of walking.

Figure 3 presents the activity of two leg muscles, the right *tibialis anterior* and the left *gastrocnemius*, and the trajectory of the center of pressure (COP) in the horizontal plane, for gait initiation and walking tasks in one subject (right-leg dominant). These recorded muscles are known to be the first to be active during gait initiation [22], starting with the activation of first swing leg *tibialis anterior* (TA) at 15±8% of our normalized phase, synchronized with the inhibition of the first stance leg *gastrocnemius* (GN). At the end of the first swing, the stance leg GN showed a burst with a peak at heel on while the swing leg TA showed a second peak at the second toe off.

During gait (Fig. 3A2), TA presented a comparable motor pattern to the one observed during gait initiation with an activity starting just after toe off on the swing leg side (right side in the example presented), presenting a first peak at mid-swing and reaching its major peak at the next toe off. A peak of GN activity was observed on the stance side just before heel on like in the first step of initiation.

The trajectory of the center of pressure (COP, Fig. 3B) was reconstructed from force plate data during gait initiation and walking trials. Each cross in the figure represents the COP value at a given percentage of gait initiation and walking (shaded areas represent double support phase). Starting from T0 point at quiet stance, the COP went backward towards the first swing leg (right here) and then forward towards the first stance leg (left here) during the preparation phase (release + unloading).

During walking, the COP is located under the stance foot during the swing phase and transfer from one foot to the other during double support (gray areas in all figures). The force developed under swing foot at heel on explains the “jumps” in COP positions. The data values presented here are in accordance with previous studies [20], [23], [24], indicating that the method we used was suitable to analyze both gait initiation and walking.

### Comparison of Erector Spinae EMG Activity during Gait Initiation and Walking

**Figure 4:**
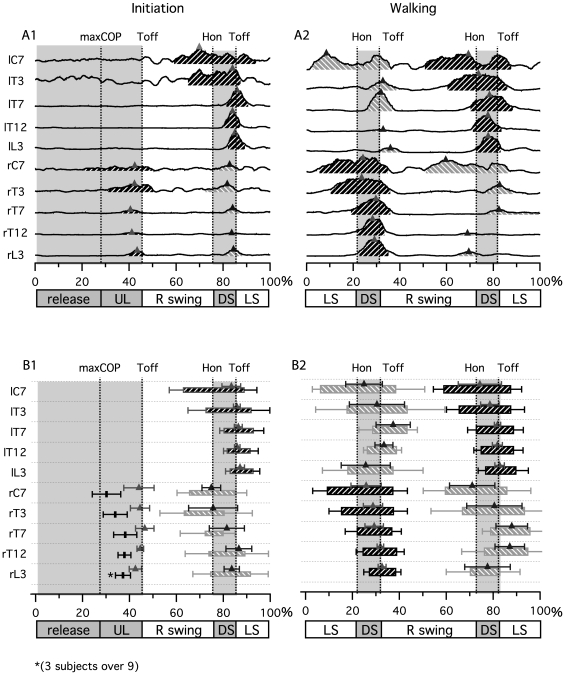
Erector Spinae (ES) muscle activity during gait initiation and gait. A) Rectified and smoothed (moving average) traces of all bilaterally recorded ES muscle from C7 to L3, for one trial and one subject during gait initiation and walking. B) Bar diagram presenting the phase of activity of ES muscle during gait initiation and walking. Each bar indicates the time during which each muscle was active. Black bars represent main activity and grey bars represent secondary activity. Small triangles represent the localization of corresponding peaks for each activity.

Figure 4A presents time-normalized ES EMG signals from one gait initiation and one walking cycle for one subject. The bar diagram in Figure 4B extends these results by reporting mean onset, peak and durations of bursts for all subjects.

During gait initiation, there was an activation of ES on the swing leg side (hatched black bursts) prior to toe off, but not on the other side (Fig. 4A1). This burst activation was propagated rostrocaudally from segment C7 to segment L3 while the corresponding peaks (triangles) were more in phase. At the end of the first swing, a second activation was observed with strong bursts that presented a descending pattern on the left side (hatched black zone), with bursts of weaker amplitude and less identifiable time organization on the right side (hatched grey zone). The bursts labeled in black were called main bursts and those labeled in grey secondary bursts.

The bar diagram in figure 4B1 summarizes these observations for all subjects. For the first main bursts (prior to the first toe off), it was not possible to precisely define their ends owing to their weak amplitude. Therefore Figure 4B1 only shows their corresponding beginnings (black lines) and peaks (triangles) at various levels. The activity started at C7 level around maxCOP (30±6%), continued at T3 level just after maxCOP (34±5%) and then at T7, T12 and L3 levels (L3 activity detectable only in 3 subjects out of 9) in the middle of the unloading phase (38±5%, 38±3% and 37±3% respectively). Linear regression analysis showed that there was a systematic and significant change in the latency with the distance (*P*<0.05) with a slope value of 0.6% per spinal level. The correspondent delay between bursts onset at C7 and L3 level during the preparation phase (release+unloading) was 10%, this correspond to 152 ms when considering the mean absolute duration of gait initiation. During the first swing, the second main ES bursts started on the stance leg side at C7 level in the middle of the swing (66±6%), continued at T3 level just before heel on (72±7%) and then at T7, T12 and L3 levels in the middle of double support (80±2%, 81±1%, 83±2% respectively). Linear regression also showed a systematic and significant change in the latency with the distance (*P*<0.05) with a slope value of 1.1% per spinal level. From C7 to L3 levels, the ES muscles onset delay was 17.5%, this correspond to 260 ms when considering the mean absolute duration of gait initiation. Corresponding peaks were synchronized with the toe off, with a short descending delay (2.6%) from C7 (83.5±4%) to L3 (87±2%) level. The corresponding burst ends occurred at the beginning of the swing phase with a lag comparable to the corresponding peaks.

During walking (Fig. 4A2), the same type of motor pattern was observed with two bursts occurring during one cycle on both sides at each muscle level. The main bursts (hatched black zone) started at C7 after mid-swing on the stance leg side (right during left swing, left during right swing), then the activity at the other axial levels started sequentially from top to bottom. The secondary bursts (hatched grey zone) presented a weaker time-organized motor pattern and they still occurred during the same period as the main activity on the controlateral side.

Figure 4B2 extends the observations to all subjects for the walking condition. The main ES activity (hatched black bars) presented a rostrocaudal metachronal pattern, like during gait initiation. The activity started at C7 after mid-swing (9±6%, 59±4%), propagated to T3 before heel on (15±4%, 65±2%), to T7 at heel on (slightly earlier than during gait initiation, 22±4%, 73±2%), to T12 after heel on (24±1%, 75±1%) and to L3 in the middle of double support (27±3%, 77±2%). Linear regression also showed a systematic and significant change in the latency with the distance (*P*<0.05) with a slope value of 1.1% per spinal level. From C7 to L3 level, the corresponding activation delay was 17.5%, this represents 175 ms when considering the mean absolute duration of gait initiation. This delay is comparable in percentage of the cycle to that observed during gait initiation (17.5%). During walking, the peaks of main bursts at C7 and T3 occurred in the middle of double support (26±6%, 29±4% and 74±9%, 79±4%), which is slightly earlier than during gait initiation. At T7, T12 and L3, they occurred at toe off, like during gait initiation. Finally, for all main bursts, the burst ends were synchronized just after toe off. The secondary bursts of ES activity (hatched grey bars) were weaker and with a variable temporal organization that did not allow precise analysis.

### Description of Trunk Kinematics

**Figure 5:**
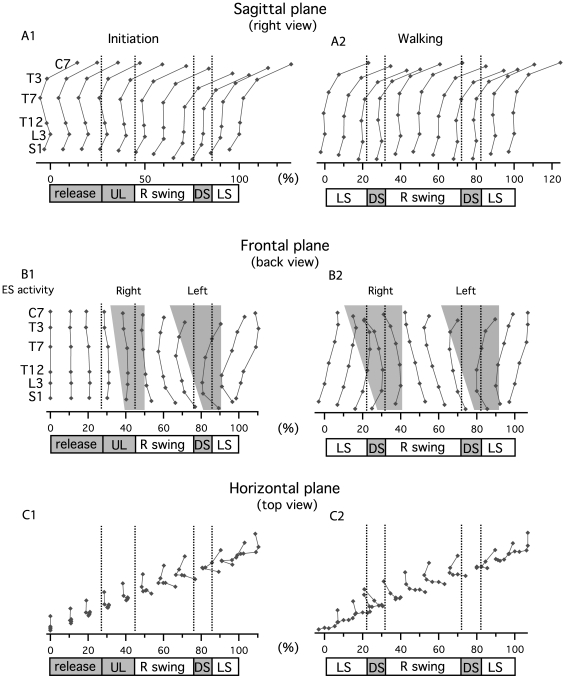
Stick diagrams of spine kinematics in the three planes: sagittal, frontal and horizontal. Each dot represents the position of one recorded vertebral level (C7, T3, T7, T12, L3, S1). Sticks are drawn for each 10% of gait initiation and walking. Bottom bar indicate phases of gait initiation and walking cycle. A) Sagittal plane. B) Frontal plane. Gray areas indicate main activity of ES muscle at each level. C) Horizontal plane.

As a first approach to analyze trunk kinematics, we plotted the mean marker positions for one subject every 10% of gait initiation phase and walking cycle in the sagittal, frontal and horizontal planes (Fig. 5).

#### Sagittal plane

During gait initiation (Fig. 5A1), there was a general inclination during the preparatory phase until the double support phase that was concomitant with a decrease in lordosis angle. During walking (Fig. 5A2), the lordosis decrease observed at the end of gait initiation persisted. Trunk inclination oscillated cyclically with the highest position occurring just before double support and the lowest one just after double support.

#### Frontal plane

During gait initiation (Fig. 5B1), there was a bending towards the side of the first stance leg (here the left side) during the preparatory phase until the middle of the unloading phase. This bending was reversed towards the side of the first swing leg (here the right side), passing through an aligned position at the transition between unloading and swing phase until the middle of the double support phase, where it reached its maximum. Finally, bending began towards the new swing leg, reaching the aligned position at 100% (ankles crossing). At the same time, the S1C7 axis presented an inclination towards the stance leg during swing and was vertical during double support when bending was maximal. During walking (Fig. 5B2), a comparable kinematic pattern was observed, with the aligned position occurring at 50% and 100% (ankles crossing) and bending towards the swing leg side reaching its maximum in the middle of the next double support phases. Interestingly, both during gait initiation and walking, the inversion of curvature occurred top-down, being initiated first in the upper trunk (thoracic area) and then in the lower trunk (lumbar area). There was a correspondance between ES muscle activities (Fig. 5B1 and Fig. 5B2) and trunk curvature. This indicated that the inclination towards one side was preceded by and concomitant with the metachronal descending ES muscle activity on the same side.

#### Horizontal plane

During gait initiation (Fig. 5C1) and walking (Fig. 5C2), there was a rotation of the thoracic region towards the swing leg, with an inversion of its direction around the double support phase. Nevertheless, this rotation followed a comparable time course to the curvature of the spine in the frontal plane. In the horizontal plane, there was also a lateral inclination of the S1C7 axis towards the stance leg during the swing phase, reaching its maximum around ankle crossing and its minimum around toe off (this can also be observed in the frontal plane).

### Angle Analysis

#### Sagittal plane

**Figure 6:**
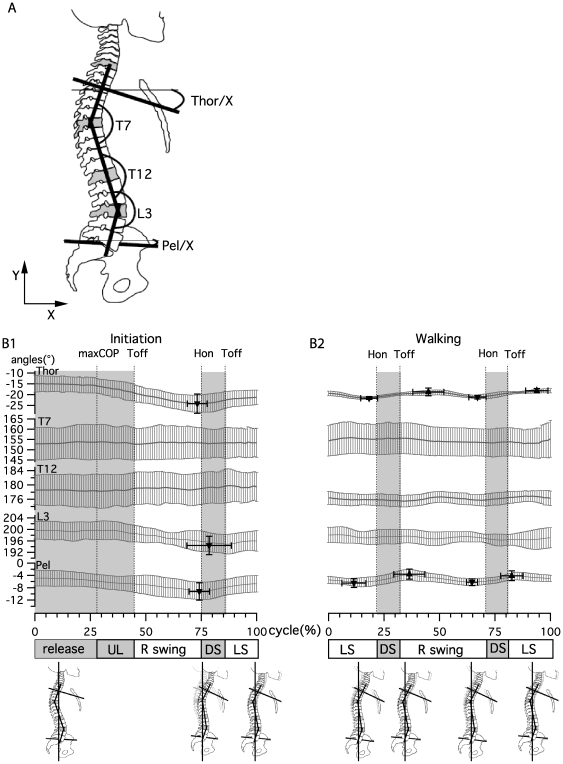
Sagittal plane kinematics during gait initiation and walking. A) Angle definition at thoracic level (Thor), T7, T12, L3 and pelvis (Pel). B) Curves present the mean±SD of angle changes for all subjects for gait initiation and walking. Triangles indicate the mean±SD of the positions and amplitudes of maxima and minima for all subjects.

The angles presented in figure 6A were defined in the plane delimited by the X and Y axis. From top to bottom these were (1) Thoracic, angle between the T3-Sternum vector and X-axis; (2) T7 angle, between T7-C7 and T7-T12 vectors; (3) T12 angle, between T12-T7 and T12-L3 vectors; (4) L3 angle, between L3-T12 and L3-S1 vectors and (5) Pelvis angle, between the vector from S1 to the middle of the superior iliac spine (SIS) and the X-axis.

During initiation (Fig. 6B1), there was an inclination of the pelvis towards the floor (mean decrease in angle of - 5±3°), reaching its maximum around heel on (74±5%). At the same time there was an inversion in the lordosis curvature (decrease in L3 angle towards 180° of - 6±3°), reaching its maximum during double support (79±10%). Mean T12 and T7 angles did not present any significant variation and exhibited a high variability due to the inter-subject variability of resting spinal angles in the sagittal plane. The thoracic segment presented an inclination towards the floor (mean decrease in angle of - 10±1.8°), reaching its maximum at heel on (73±4%). The thoracic segment inclination was therefore mainly supported by the pelvis inclination and the inversion in the lordosis curvature.

During walking (Fig. 6B2), the pelvis angle underwent two small oscillations (3±1° peak to peak amplitude) around the position reached at the end of initiation. It was more inclined at the end of the swing phase (11±5% and 65±3%) and less at the beginning of the swing phase (37±7% and 83±5%). At the thoracic level there was an oscillation of the same amplitude (3±1°), with comparable timings for maximal (18±4% and 67±4%) and minimal (45±5% and 94±5%) inclination. At L3, T12 and T7, angular variations were small. This was in accordance with a parallel oscillation of inclination in the pelvis and thorax.

#### Frontal plane

**Figure 7:**
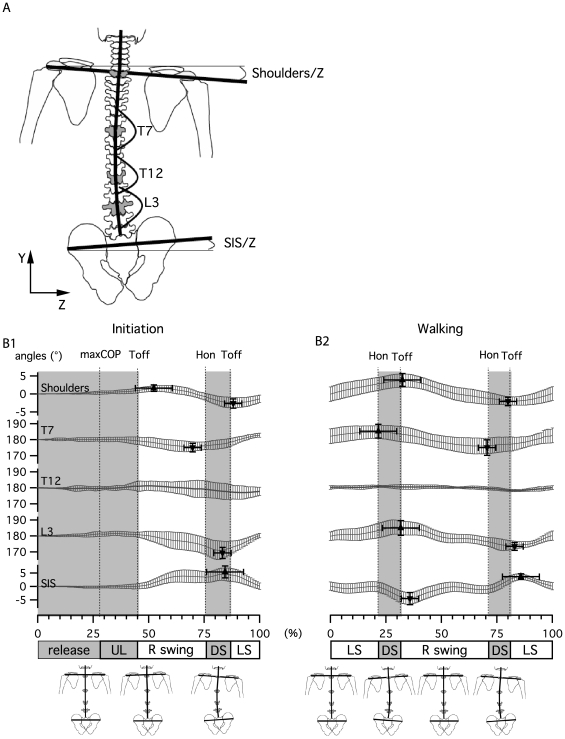
Frontal plane kinematics during gait initiation and walking. A) Angle definition at shoulder level, T7, T12, L3 and superior iliac spines (SIS). B) Curves present the mean±SD of angle changes for all subjects for gait initiation and walking. Triangles indicate the mean±SD of the positions and amplitudes of maxima and minima for all subjects.

The angles presented in figure 7A were defined in the plane delimited by the Y and Z axis. From top to bottom these were: (1) shoulder angle, between the left-right shoulders vector and Z-axis; (2) T7 angle, between T7-C7 and T7-T12 vectors; (3) T12 angle, between T12-T7 and T12-L3 vectors; (4) L3 angle, between L3-T12 and L3-S1 vectors and (5) superior iliac spine (SIS) angle, between the left-right SIS vector and the Z-axis.

During gait initiation (Fig. 7B1), the first trunk activity was a slight inclination of the shoulders (1.1±0.85°) towards the first stance leg (left side here), which reached its maximum after the first toe off (52±8%). This activity was followed by an inclination in the opposite direction (towards the first swing leg) of the spine at T7 and L3 that reached its maximum at 70±5%, just before heel on for T7 and at 83±5% just before toe off for L3. The phase shift in curvature inversion between the T7 and L3 angle was 13% of gait initiation. At T12, the angular change did not present any significant variation. The first SIS inclination followed the curvature of the spine to reach its maximum at 84±3% concomitant with an opposite inclination of the shoulders at 88±4%. At the end of initiation (ankles crossing), shoulders and SIS were horizontal while the vertebrae were aligned (T7, T12 and L3 at 180°).

During walking (Fig. 7B2), the pattern was comparable to that observed at the end of initiation, with the SIS and shoulders maintaining the phase opposition observed at the end of gait initiation, and extreme inclinations occurring at toe off (32±5% and 80±8% for shoulders, 35±1% and 86±2% for SIS respectively). The phase shift between T7 and L3 angles remained, the first reaching its extremes at heel on (22±6% and 71±7%) and the second at toe off (32±3% and 83±4%). The lag values between T7 and L3 were 10% and 12%. At T12 the activity was too weak in the frontal plane to be accurately analyzed during walking. At 0%, 50% and 100% (ankles crossing), the shoulders and SIS were parallel while the vertebrae were aligned (T7, T12 and L3 at 180°).

#### Horizontal plane

**Figure 8:**
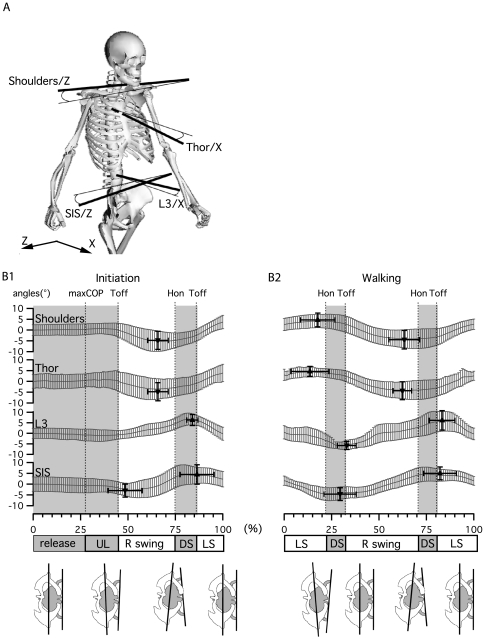
Horizontal plane kinematics during gait initiation and walking. A) Angle definition at shoulder level, thoracic level (Thor), L3 and superior iliac spines (SIS). B) Curves present the mean±SD of angle changes for all subjects for gait initiation and walking. Triangles indicate the mean±SD of the positions and amplitudes of maxima and minima for all subjects.

The angles presented in figure 8A were defined in the plane delimited by the X and Z axis. From top to bottom these were: (1) shoulder angle, between the left-right shoulders vector and Z-axis; (2) thoracic angle, between T3-Sternum vector and X-axis; (3) L3 angle, between the sum of L3T12 and L3S1 vectors (delimiting the lumbar plane) projection on horizontal plane and the X-axis; (4) superior iliac spine (SIS) angle, between the left-right SIS vector and the Z-axis.

During gait initiation (Fig. 8B1), the first activity was a rotation of the pelvis towards the first swing leg (the right side in our protocol), the rotation in the other direction being initiated at toe off (49±9%), together with a rotation of the lumbar area in the same direction and an inverse rotation of the upper trunk (thoracic area and shoulders). The maximum rotation presented a phase shift at the end of the swing phase with a change in the upper trunk direction before heel on (67±5% for shoulders and 66±5% for thorax), and in the lower trunk at toe off (84±3% for lumbar area, 86±5% for pelvis).

During walking (Fig. 8B2), the opposite rotations of the upper and lower trunk remained as at the end of gait initiation, with a comparable phase shift in rotation inversions before heel on for the upper trunk (18±9% and 63±8% for shoulders, 14±10% and 62±5% for pelvis) and at toe off for the lower trunk (33±5% for 83±7% lumbar area, 30±9% and 82±8% for pelvis). In comparison with the frontal plane (Fig. 7), there were similarities between trajectories of the upper and lower trunk in the horizontal and frontal planes, since the upper trunk inverted its trajectory around heel on in both cases, while the lower trunk did so around toe off.

### Comparison of Kinematic and EMG

**Figure 9:**
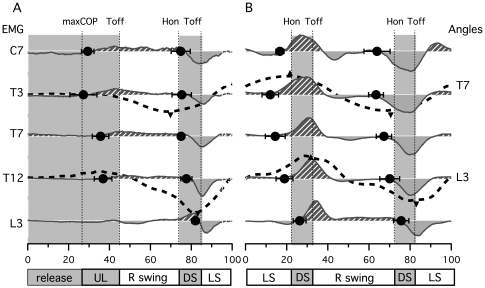
EMGs versus kinematics. Plain curve represents the difference between right and left normalized EMG at C7, T3, T7, T12, L3 and S1 levels. Filled circle indicates the mean±SD of curve zero crossing instants for all subjects, i.e. the time at which the side of strongest ES muscle activity changes. Dotted curves represent frontal angles of the spine at T7 and L3 level. The triangles indicate the corresponding maxima (as in figure 7) and represent the inversions of curvature directions. A, during gait initiation and B, walking.

In order to illustrate the asymmetric activation of ES muscle during gait initiation and walking and establish their possible link with trunk kinematics (Fig. 9), we used a method based on the mean difference (right side minus left side) between normalized EMG signals of ES muscle at each level (C7, T3, T7, T12 and L3). ES muscle bursts were normalized in amplitude under each condition, with the maximum of the first walking cycle for gait initiation and with the maximum of each cycle for walking. The signal obtained could be seen as reflecting the strength ratio between right and left ES muscle. The resultant wave was positive (hatched under curve) when the right side EMGs were stronger and negative (filled over curve) when the left side EMGs were stronger. The filled circle on each trace indicated the time (mean±SD) at which the signal was inverted. This indicated at each level when the strength ratio between the two lateral muscles was inverted. Angles at T7 and L3 in frontal plane were also plotted (dotted curves) against EMG differences at T3 and T12 respectively according that those muscles can be responsible of trunk lateral flexion. The small triangle represents the mean times of angle maxima. This indicated an inversion of movement for the angles. The comparison of strength ratio in EMG and angles in kinematics shows that the curvature inversion of the spine is supported by an inversion of strength ratio toward the corresponding side.

## Discussion

In the present study we examined the activity of ES muscles during gait initiation and compared it to what occurs during ongoing locomotion in order to gain further insight in their pattern. Furthermore, we correlated the activation of back muscles with the different kinematic and dynamic phase events in both conditions to determine the effect of ES muscles and their role.

### EMG vs Kinematics and Dynamics

In a recent study, we showed that the ES muscle expresses a double burst at each spinal level during walking in humans[12], extending the results of other authors who reported double bursts of activity at the lumbar level [5], [13], [14]. One of these bursts has a highest amplitude and propagates rostro-caudally [12]. Our present results confirm the propagation of the main burst along the spine not only during walking but also during gait initiation. Furthermore, we established the spatiotemporal relationship between ES activation and the main kinematics and dynamic events that occur during gait initiation and walking. It therefore appears that ES muscle activation occurs before leg lifting on the same side (i.e. right main ES main burst precedes right swing, Fig. 4), propagating through the back from C7 to L3. Burst then terminates synchronously at each level at the beginning of the swing.

To our knowledge, trunk kinematics has not been clearly reported during gait initiation. In the sagittal plane, at the first toe off, the pelvis and lumbar area moves the trunk in a general forward inclination, to reach its maximum at first heel on (Fig. 6). During walking, the trunk maintains this inclination and oscillates around this position twice per cycle [11], [25]. Those oscillations in the saggital plane are mainly pelvic in origin. Nevertheless the phase switch between thoracic and pelvic oscillations (Fig. 6) provides evidence for the occurrence of intersegmental oscillations in the sagittal plane, again as reported in other papers [26], [27]. During gait initiation in the frontal plane, spinal curvature begins around the first toe off. Immediately after, the kinematic pattern of the spine is very much like that observed during walking, expressing a curvature toward the last swinging leg (Fig. 7). This curvature is mainly localized in the lumbar and upper thoracic area, the middle segment conserving its straightness when walking, as reported in other studies [26], [27]. The shoulders and pelvic inclination each present one oscillation, with only a slight delay in the transition to the opposite phase (Fig. 7). In the horizontal plane, a comparable pattern is observed for gait initiation and walking with upper and lower trunk rotation occurring as a single oscillation per cycle, which is almost opposite in phase (Fig. 8). In this plane, the center of rotation between thoracic and pelvic belts is presumably positioned between L3 and T7, as shown in [28]–[30].

**Figure 10:**
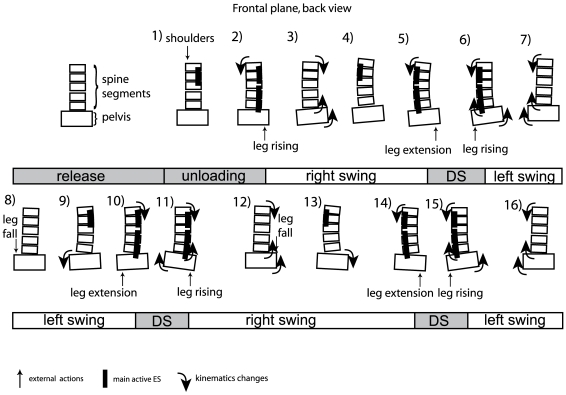
Cartoon of trunk activity. Each picture is a schematic representation of trunk movements and activities at a given time during gait initiation phase and walking cycle.

The ES muscles are known to act as erector of the rachis when activated bilaterally but also as lateroflexors when activated asymmetrically. Using biomechanical observations, some authors also reported that the rotation of vertebral segments may be induced by lateral bending [30]–[32] and that the two phenomena are closely linked during gait [10]. As previously reported [33], the observed spine angulations in the frontal plane result both from spinal lateroflexion and from rotation of the kyphosis and lordosis curvature. Our protocol with only one marker at each studied vertebral level did not allow us to discriminate the involvement of each phenomenon. Nevertheless, in the frontal and horizontal planes, since similar movement direction inversions occurred, with the trunk acting synchronously in these two planes (Fig. 7, 8), then comparing ES activity with trunk kinematics in the frontal plane is relevant. However a more detailed protocol with more markers for each studied vertebral segment would allow a more precise three-dimensional monitoring of spinal kinematics [34]. Figure 10 presents a simplified model of trunk kinematics that summarizes our observations on the action of ES muscle in the frontal plane. A series of schematic representations of trunk muscles activity and simplified external actions on the pelvis are presented. During gait initiation, at maxCOP the activity of right ES precedes a curvature of the trunk toward the right side and an elevation of the pelvis above the right leg (Pictures 1–2). The spinal curvature then increases until upper ES muscles activation on the opposite left side (Picture 3–4), which triggers an inversion in the upper trunk curvature at heel on (Pict 5), followed in the lower trunk at toe off after lower ES muscle activation (Pict 6). During double support, the pelvis rises towards the right due to the extension of the next stance leg until toe off (Pict 5–6). After toe off, at the offset of ES activity on the same side, the pelvis rises on the swing leg side while the trunk continuse to bend downward in the opposite direction (Pict 7). A new step then starts on the other side with the trunk bending in the other direction (Fig. 10, pict 8–16). The ES muscles thus seem to contract concentrically so as to invert the curvature (i.e. that provokes curvature toward the other side) of firstly the upper then the lower trunk. Such a metachronal organization of ES muscle activation seems to be adequate for a proximo-distal action that would help the pelvis and the swing leg elevation to effect the step, with the upper trunk used as inertial reference.

### Motor Program Organization

The existence of a central pattern generator (CPG) for limb locomotion has been well established in animals. The possibility that CPGs for locomotion also exist in humans has been recently reviewed [35]–[38], and reciprocal interactions between arm and leg movements have been established [39]–[41]. More recently, a phylogenic comparison of axial neuronal network functioning suggests that despite dramatic changes undergone by the human muscular and skeletal systems during the switch from quadrupedal to bipedal walking and erect posture, common mechanisms for trunk control may be shared throughout vertebrates [42]. As mentioned previously, our data show (1), that the ES muscles are active in a rhythmic pattern even prior to first toe off during gait initiation, thereby pointing to centrally programmed activity; (2), the ES muscle seems to drive trunk movements rather than reacting to them, which suggests an anticipator control of the trunk by a dedicated CPG. Indeed, since anticipatory actions are known to be much more efficient than reactive responses [43], then rhythmic trunk activity as observed is likely to be more efficient if programmed like a CPG.

Our main interest in studying gait initiation was to determine how the switch from a postural to a dynamic state occurs and identify the motor programs involved in this transition. During gait initiation, the leg motor pattern for the *tibialis anterior* (TA) and *gastrocnemius* (GN) as well as the COP trajectory were similar in our study to those reported in other studies [19], [20], [22], [44], [45]. It has been proposed that the gait initiation program starts with the activation of TA and inhibition of GN muscle, thereby inducing a “fall forward” phenomenon [20], [22], [45], [46]. The termination of this program and the switch to the walking program may occur at first heel on as reported by Brunt et al. (1991). Our results show that the ES muscles present a comparable motor pattern during gait initiation and walking from before the first toe off (Fig. 4). In kinematics, the similarities between gait initiation and walking begin at first toe off in the frontal and horizontal plane and around the first heel on in the sagittal plane (Fig. 5, 6, 7, 8). Except in the sagittal plane, trunk activity was comparable to a half walking cycle from the middle of gait initiation. Data from force measurements show that during gait initiation the peak of force under the first moving foot at maxCOP is similar to that observed of the next swing foot at toe off during walking (Fig. 2). Altogether these data suggest that the end of gait initiation occurs at first heel on and that the walking program begins before first toe off. A likely hypothesis therefore is that the motor programs for the transition from gait initiation to walking are not sequentially activated, but that the walking program onset before the end of the gait initiation program acts like in a fuzzy logic controller [47], which is known to be one of the best ways to command an inverted pendulum [47]. The walking program might begin around maxCOP, while the initiation program could end at heel on. The principle of the walking motor program beginning in a propulsive phase is coherent with observations made in other studies [48] from muscle activation patterns at different walking speeds, situating the origin of walking cycle generation in propulsion rather than correlated to the heel strike event.

### Validity of Cycle Definition

To compare gait initiation and steady-state walking we used an original cycle paradigm. A walking cycle may be defined by the interval that separates the same two consecutive events. Usually the walking cycle is arbitrarily defined by taking successive contacts of the same heel. In our study we defined the cycle between two ankle crossings as described in the Results section, a choice that allowed a direct comparison to be made between gait initiation and walking. Gait initiation is the transition from an upright posture (shoulders and pelvis aligned in frontal and horizontal planes) to a bending one. During gait initiation, this transition is located around the first toe off (corresponding to ankle uncrossing), depending on the segment considered. During the gait cycle, the transition from straightness to bending on one side of the trunk in the frontal and horizontal plane occurred around the middle of the swing phase [Fig. 7, 8, [49], [50]]. This also corresponds to “ankle-crossing” in the direction of movement.

Finally the information on gait cycle phase based on ankles interval is continuous and may be used as a reference at each moment during the cycle, while that provided by footswitches is discrete. In future studies, we will be able to compare different modes of locomotion like walking, running and cycling with a consistent cycle definition.

In conclusion, we have shown in the present study that trunk movement during gait initiation and walking share similarities: (1) a metachronal activation of ES muscle occurs during the preparation of the first step for gait initiation as well as just before the double support phase during walking, suggesting that this organization corresponds to an anticipation to propulsion, and probably controlled by a CPG; (2) this muscle activity occurs just before a kinematic activity in a corresponding direction, suggesting a direct relationship between the two.
